# Synergistic Delamination Toughening of Glass Fiber-Aluminum Laminates by Surface Treatment and Graphene Oxide Interleaf

**DOI:** 10.1186/s11671-020-03306-z

**Published:** 2020-04-07

**Authors:** Xiaopeng Wu, Huiming Ning, Yaolu Liu, Ning Hu, Feng Liu, Shu Wang, Kaiyan Huang, Yudu Jiao, Shayuan Weng, Qiang Liu, Liangke Wu

**Affiliations:** 1 College of Aerospace Engineering, Chongqing University, 174 Shazheng St., Shapingba District, Chongqing, 400044 People’s Republic of China; 2 Chongqing Key Laboratory of Heterogeneous Material Mechanics, Chongqing, 400044 People’s Republic of China; 3 State Key Laboratory of Advanced Design and Manufacturing for Vehicle Body, Changsha, 410082 People’s Republic of China; 4 State Key Laboratory of Reliability and Intelligence Electrical Equipment, Hebei University of Technology, Tianjin, 300401 People’s Republic of China; 5 School of Mechanical Engineering, Hebei University of Technology, Tianjin, 300401 People’s Republic of China; 6 AVIC Composite Technology Center, Beijing, 101300 People’s Republic of China

**Keywords:** Glass fiber-aluminum laminates, Interlaminar fracture toughness, Graphene oxide, Surface treatment, Synergistic toughening

## Abstract

The synergistic effects of surface treatment and interleaf on the interlaminar mechanical properties of glass fiber-aluminum laminates were studied. Aluminum sheets were treated with alkaline etching. Meanwhile, a graphene oxide (GO) interleaf was introduced between the aluminum sheet and the glass fiber-reinforced epoxy composite. Double cantilever beam and end-notched flexure tests were employed to evaluate the interlaminar fracture toughness of the glass fiber-aluminum laminates. The obtained results show that the toughening efficiency of the interleaf is dependent on the aluminum surface characteristics as well as the GO loading. Further comparison reveals that the highest mode-I and mode-II fracture toughnesses are obtained in the specimens with alkali etching treatment and addition of GO interleaf with 0.5 wt% of GO loading, which are 510% and 381% higher in comparison to the plain specimen. Fracture surfaces were observed to further uncover the reinforcement mechanisms.

## Introduction

Fiber metal laminates (FMLs) are a novel type of hybrid lightweight composites, which are composed of metal substrates and fiber-reinforced plastics (FRPs) [1]. Due to the hybrid structure, FMLs provide excellent mechanical properties including high specific strength and stiffness, good fatigue resistance, and excellent damage tolerance [2, 3]. However, weak interfacial bonding of FMLs may cause delamination and debonding failure due to the physical property differences between the metal sheet and composite layer [4]. Therefore, it is essential to improve the interlaminar mechanical properties for FMLs.

To improve the interlaminar mechanical properties of FMLs, a series of surface treatment methods, such as acid or alkali etching [5–7], anodizing [8], laser ablation [9, 10], silane coupling agent treatment [11, 12], and atmospheric pressure plasma [13], have been proposed to modify the surface morphology of the metal sheets. Among these methods, alkali etching is considered a simple and efficient method, which can remove the weak native oxide layer on the metal surface as well as create a rough surface and stable oxide layer to strengthen the interfacial bonding. Nowadays, the development of nanotechnology has significantly expanded the application domain of nanomaterials in various fields including aerospace [14], electronic device [15], energy [16], and environment [17]. Incorporating nanomaterials into the interlaminar layer is another effective way to improve the interlaminar properties of laminated composites by modifying the rich-resin region. Common interleaf nanomaterials, such as nanoclay [18], vapor grow carbon fiber [19], and carbon nanotube [20], have been widely used in FMLs.

Graphene, consisting of a single layer of carbon atoms, exhibits ultrahigh mechanical [21], electrical [22], and thermal [23] properties, which make it a promising candidate for modifying the polymer matrix. Rafiee et al. [24] fabricated the epoxy resin-based bulk composites reinforced with graphene by solution blending. The results demonstrate a 40% and 53% increase in the tensile strength and fracture toughness of the nanocomposites, respectively. Kostagiannakopoulou et al. [25] adopted graphene as toughening agents in matrix to prepare carbon fiber-reinforced polymers and observed a 50% increase in the interlaminar fracture toughness. However, the toughening efficiency of graphene depends on the dispersion state of graphene in polymer matrix. The surface chemical properties of graphene affects its interfacial compatibility with polymer matrix and then leads to a poor dispersion of graphene [26]. As a derivative of graphene, graphene oxide (GO) contains various oxygen-containing groups (hydroxyls, epoxide, carbonyl, and carboxylic) on its surface, which endow it a better dispersion and compatibility in the polymer matrix compared with graphene. Owing to its potential advantages, GO has emerged as an effective reinforcement in polymer composites [27–29]. A significant increase of mode-I interlaminar fracture toughness of 170.8% has been reported for carbon fiber laminates modified with graphene oxide interleaf [30]. Pathak et al. reported a comprehensive improvement in flexural modulus, flexural strength, and interlaminar shear strength of carbon fiber composites by incorporating 0.3 wt% GO [31]. However, to the best of our knowledge, the interlaminar mechanical properties of FMLs toughened by GO interleaf has not been studied to date. Furthermore, the synergistic effects of the surface treatment of metal plate and GO interleaf have not been well understood.

The FMLs studied in this paper are based on glass fiber-aluminum laminates (GFRP/Al laminates) which have been widely used in various fields, such as aerospace and automobile industries. By combining alkali etching treatment and GO-reinforced epoxy interleaf, the mode-I and mode-II interlaminar fracture toughnesses of the GFRP/Al laminates were systematically investigated. In addition, various characterizations were carried out to uncover the synergistic toughening mechanism.

## Methods/Experimental

### Materials

Natural graphite flakes (XF051, 100 mesh) bought from Nanjing XFNANO Materials Tech Co., Ltd., were used to prepare graphene oxide by the modified Hummers’ method [32]. The epoxy adhesive used in this study was diglycidyl ether of bisphenol F. EPON862. Polyamide (Epikure3140A) was chosen as the curing agent. Al alloy (7075) plates with a thickness of 2.5 mm were selected as the metallic part of the FMLs. Unidirectional glass fiber prepregs (GFRP prepregs) were provided by Weihai Guangwei Composite Material Co., Ltd, China. All other materials, such as sodium hydroxide (NaOH), *N*,*N*-dimethylformamide (DMF), acetone, hydrochloric acid (37 wt%), and chromium trioxide, were supplied by Chengdu Kelong Chemical Reagent Co., Ltd. (China).

### Specimen Preparation

**Fig. 1:**
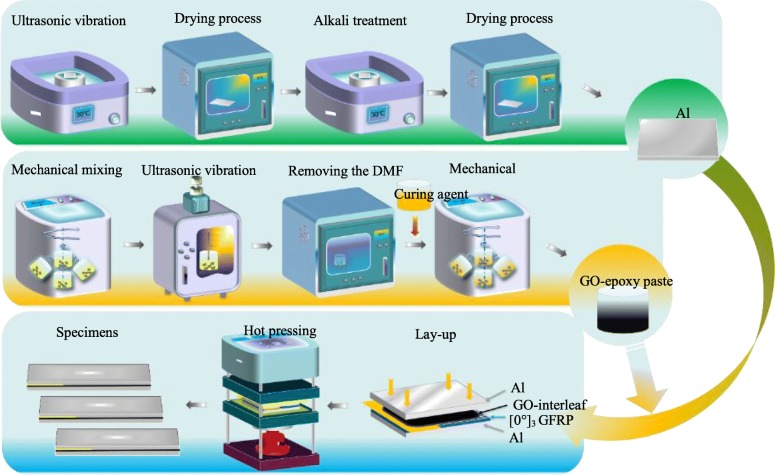
Schematic illustration of specimen fabrication and hot pressing process

The fabrication of GFRP/Al laminates is schematically shown in Fig. 1. First, the surface treatments of the aluminum plates were carried out in the following steps: (a) the Al plates were rinsed with acetone for degreasing and then dried in an oven to remove the moisture, (b) the Al plates were immersed in 0.1 M NaOH solution and ultrasonicated for 30 min at ambient temperature to modify the surface morphology of the Al plates, (c) the treated Al plates were taken out and ultrasonicated in distilled water until the reaction of aluminum with NaOH was terminated, and (d) the washed Al plates were dried at 60 °C for 1 h . More details about the surface treatment by alkali etching can be found in Ref. [5].

Then, GO-reinforced epoxy paste was fabricated as the toughening interleaf. The detailed preparation process of the GO interleaf is similar to that in our previous work [30]. The main steps include (a) preparing GO/DMF suspension by mechanical mixing and ultrasonic vibration, (b) pouring epoxy resin into the GO/DMF suspension and mixing them by planetary string and ultrasonication, (c) heating the above mixture for removing the DMF, and (d) adding the curing agent under constant stirring.

Finally, the FMLs were prepared using the hot pressing method as shown in Fig. 1. The main steps include the following: (a) Three piles of unidirectional GFRP prepregs were stacked between two pieces of aluminum plates through a lay-up process. During the manufacturing process, the obtained GO-epoxy paste was carefully smeared at the interface of aluminum plates and GFRP prepregs using a blunt blade, where the area density of epoxy was set to a constant value of about 167 g/m^2^. (b) A release film was inserted to make an initial crack. (c) The FMLs were packed by a polyimide film and cured based on the temperature of 130 °C and the pressure of 0.12 MPa.

To explore the effects of the surface treatment and GO-epoxy interleaf on the fracture toughness of the FMLs, five types of specimens were prepared, i.e., the plain, GO0.5%, SH-GO0%, SH-GO0.5%, and SH-GO1%, where “SH” denotes the alkali etching treatment of Al plates, “GO” represents GO-epoxy interleaf, and the percentage after “GO” denotes the weight fraction of GO in the epoxy.

### Experimental Tests and Characterization

**Fig. 2:**
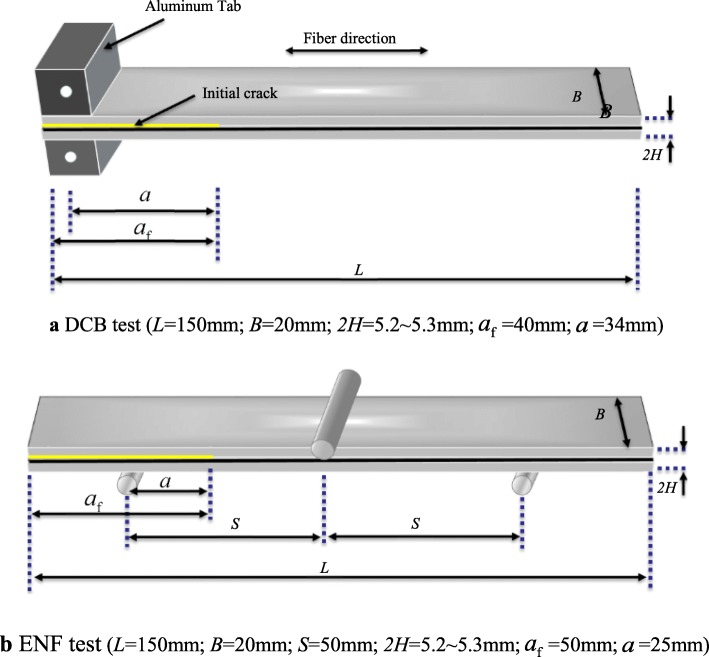
Schematic illustration of the specimen for **a** DCB and **b** ENF test

Double cantilever beam (DCB) and end-notched flexure (ENF) tests were carried out to measure the mode-I and mode-II interlaminar fracture toughness of the GFRP/Al laminates according to Japanese Industrial Standard (JIS) K7086 [33]. The configurations of DCB and ENF specimens are shown in Fig. 2. The detailed test procedures and the fracture toughness calculation methods are similar to those in Ref. [33].

The surface morphologies of the graphite flakes, GO, aluminum sheet, and fracture surfaces of the tested specimens were characterized by scanning electron microscopy (SEM). Meanwhile, the nanostructures of the GO sheets were observed by transmission electron microscopy (TEM) and atomic force microscopy (AFM). The chemical structure of GO and aluminum substrates were characterized on an ESCALAB 250Xi XPS system (Thermo Electron Corporation, USA). In addition, the surface roughness and wettability performance of the aluminum sheet were studied using optical interferometric profiler and contact angle goniometer, respectively.

## Results and Discussion

### Characterizations of the GO

**Fig. 3:**
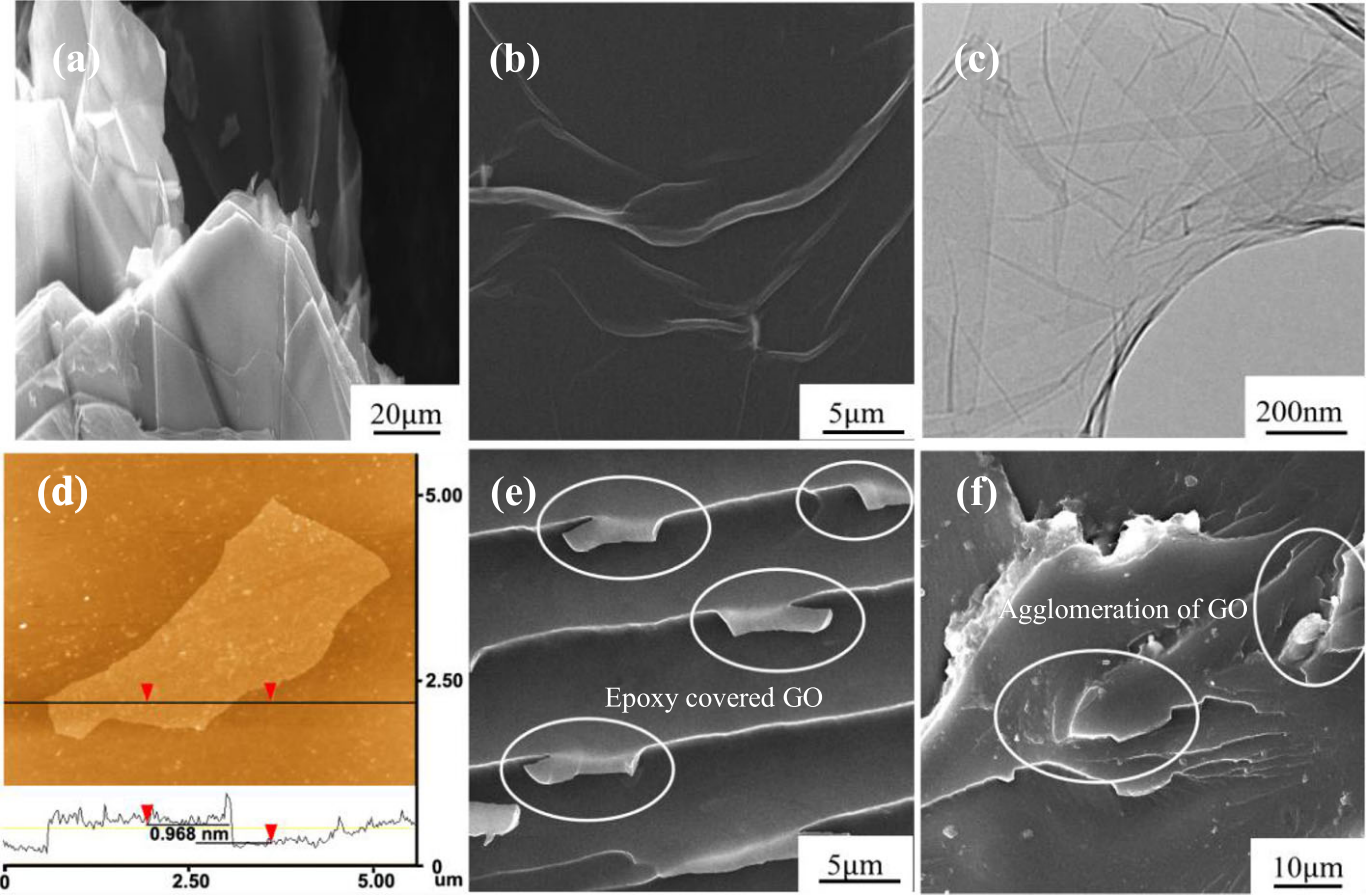
SEM images of **a** graphite flakes. **b** GO sheets. **c** TEM image of GO. **d** AFM image of GO. **e** GO sheets in epoxy resin (0.5 wt%). **f** GO sheets in epoxy resin (1.0 wt%)

The surface morphologies of graphite and GO flakes were characterized by SEM and TEM as shown in Fig. 3. It can be observed a multi-layer structural of graphite flake in Fig. 3a, while the SEM and TEM images of GO in Figs. 3b and c exhibit a thin-layer structure. This indicates that the multi-layer structure of graphite is stratified and graphene oxide has been successfully synthesized. Figure 3d presents the AFM image of the GO nanosheet. The thickness of the prepared GO is about 0.968 nm, which indicates that a single layer of graphene oxide nanostructure has been achieved after a complete exfoliation from the graphite. In addition, the dispersion state of GO plays a crucial role in the toughening of polymers. A poor dispersion of GO may result in unfavorable effects on the transition of stress from the resin to GO nanosheets. Therefore, the dispersed GO needs to be characterized and evaluated. Figures 3e and f show the microstructures of GO sheets after dispersion in epoxy resin. Incorporation of GO at a concentration of 0.5 wt% exhibits a good dispersion in the resin, while slight aggregation of GO can be observed at a higher concentration (1.0 wt%), which may result in stress concentration and therefore weaken the strength and toughness of the epoxy.

**Fig. 4:**
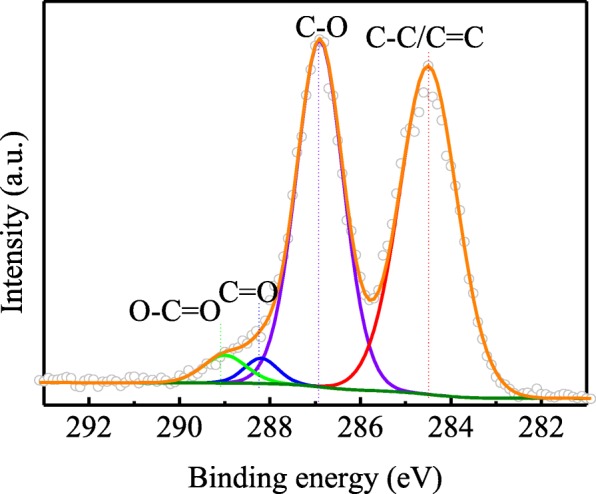
XPS C1s spectrum of the GO sheets

The chemical structure on the surface of GO is another important factor that affects the toughening efficiency of GO in the polymers, which is responsible for the interfacial interaction between GO and the resin matrix [30, 31, 34]. X-ray photoelectron spectroscopy (XPS) was employed to identify the surface chemical property of the prepared GO. As shown in Fig. 4, the C 1s spectrum of GO is split into four peaks which are assigned to four types of carbon bonds: (1) C–C/C=C (284.5 eV), (2) C–O (286.9 eV), (3) C=O (288.2 eV), and (4) O–C=O (289 eV) [35]. The presence of oxygenated functional groups is beneficial to the dispersion of GO and bonding strength between GO and the polymeric matrix [30, 31, 34]

### Physical and Chemical Properties of Aluminum Alloy Surface

In general, interfacial debonding between the FRP composite and metal involves both interfacial and cohesive failure, which is commonly affected by the surface characteristics of the metal sheet. Therefore, the physicochemical properties including the surface microstructure, roughness, chemical composition, and wettability of the aluminum alloy surface were characterized by various measuring instruments.

**Fig. 5:**
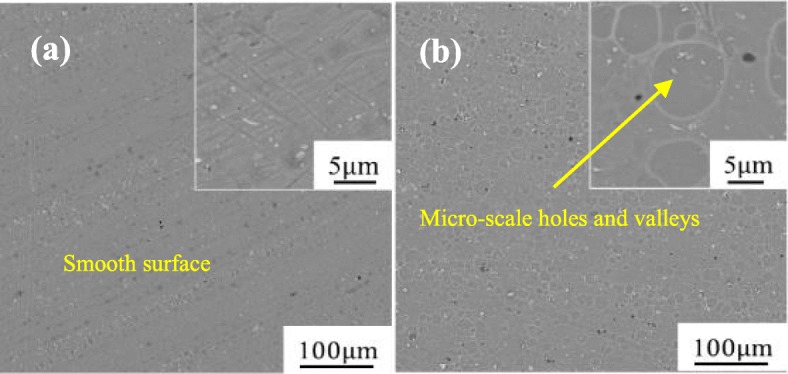
SEM images of the Al surface after **a** degreasing and **b** alkali etching

Figure 5 shows the surface morphology and microstructure of the Al alloy plates before and after alkali etching. As can be seen, the Al alloy surface treated by alkali etching becomes rougher than that of the degreasing Al alloy surface. Many micro-scale holes and valleys can be observed on the surface of the Al alloy treated by alkali etching, which are favorable for the filling up of epoxy resin and GO to form mechanical interlocking and enhance the bonding strength of the composite/metal interface [7, 19, 36]. In addition, the surface profiles of the Al alloy plates before and after alkali etching were also measured using the optical interferometric profiler. The corresponding surface roughness values (*R*_*a*_, *R*_*q*_, and *R*_*z*_) are summarized in Table 1, where *R*_*a*_ represents the arithmetic average deviation of the profile, *R*_*q*_ is the root mean square roughness and *R*_*z*_ represents the ten-point height of irregularities. A significant difference in measured values before and after alkali etching can be observed from Table 1, which is consistent with the SEM observation results in Fig. 5. The high roughness of the alkali etching surface implies an increase in the specific surface area which is beneficial for the mechanical interlocking between the Al alloy sheet and the polymer matrix.

**Table 1:** Surface roughness of the Al surface after: (a) degreasing and (b) alkali etching

Surface treatment	*R*_*a*_(μm)	*R*_*q*_(μm)	*R*_*z*_(μm)
Degreasing	0.10	0.14	0.74
Alkali etching	0.42	0.58	2.62

**Fig. 6:**
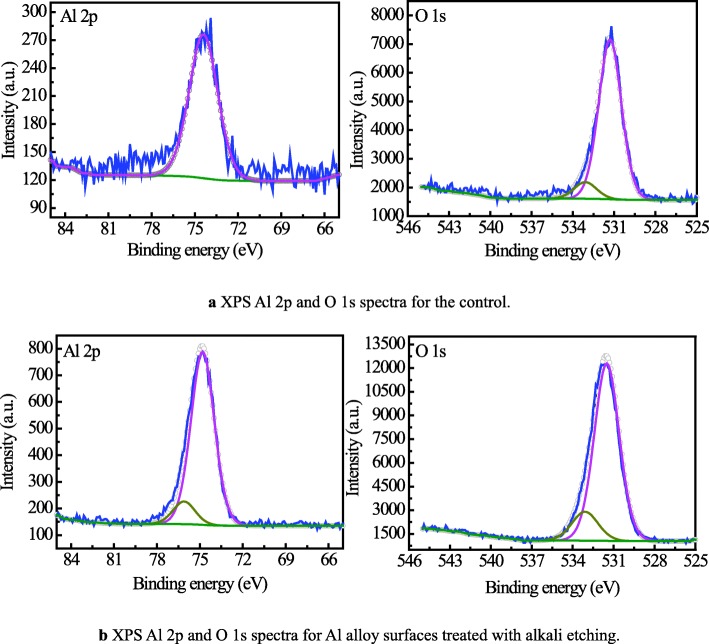
Narrow scan of XPS spectra of Al alloy surface

XPS was performed to analyze the chemical modification of Al alloy surface with different surface treatments. Figure 6 presents the narrow scan spectrum of Al 2p and O 1s for the un-etched and etched Al alloy surfaces. Figure 6a shows the de-convoluted Al 2p ionization spectra of un-etched surfaces, which only has one peak with a binding energy of 74.4 eV corresponding to γ-aluminum oxides (γ-Al_2_O_3_) [37]. The O 1s spectra of the un-etched surface is split into 2 peaks, which are assigned to Al_2_O_3_ (531.3 eV) and aluminum hydroxide (533.1 eV), respectively [13].

Figure 6b shows the de-convoluted Al 2p ionization spectra of the etched surface, where the first peak located at 74.8 eV is associated to Al_2_O_3_, and the peak at 76.1 eV corresponds to aluminum hydroxide [38]. The O 1s spectra of the etched surfaces show two peaks, one for Al_2_O_3_ (531.5 eV) and the other for aluminum hydroxide (533.1 eV) [13]. Comparing the results of the un-etched and etched Al alloy surface, a shift in the binding energy of Al 2p implies the surface chemical property of the Al alloy has been changed by the surface treatment [6]. Meanwhile, hydroxide to oxide intensity ratios of the O 1s peak of the etched surfaces is higher than that of the un-etched surfaces, which could improve the interfacial adhesion due to the formation of more hydrogen bonds between the hydroxyl groups on aluminum hydroxide and epoxy molecules [13].

**Fig. 7:**
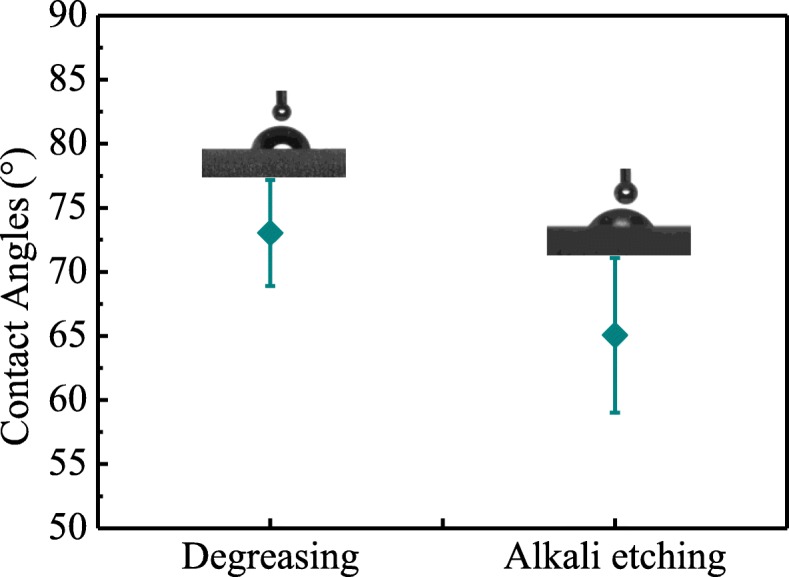
Contact angle of water droplet on the Al alloy surface with different surface treatments

To investigate the effect of surface treatment on the wettability of the Al alloy surface, standard droplets were dropped onto the surface of the tested samples to measure the contact angles. Figure 7 presents the image of static contact angles for the Al alloy surface before and after alkali etching. It can be found that the surface of the alkali-treated Al plate has a smaller contact angle, which implies the better wettability of the Al alloy surface with the alkali etching treatment. The increased wettability may also contribute to the improvement of the interfacial bonding strength [6].

### Mode-I Interlaminar Fracture Toughness

**Fig. 8:**
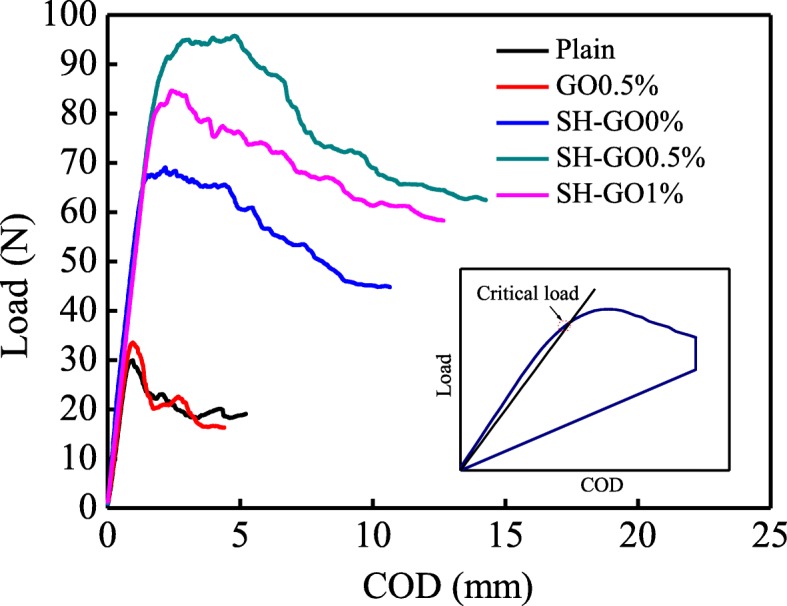
Representative load and crack opening displacement (*P*-COD) curves for different specimens during DCB tests

DCB tests were conducted on different types of GFRP/Al laminates. Figure 8 shows the relationship between the load *P* and crack opening displacement (COD). It can be found the overall tendency in load and crack opening displacement (*P*-COD) response of the FMLs specimens is almost similar, i.e., the applied load firstly increases linearly, and then increases slightly in a nonlinear pattern until the load reaches the maximum, followed by a gradual decline in the final stage. Due to the uncertainty of the crack growth initiation, the critical load (*P*_C_) is defined as the intersection of the *P*-COD curve with a line corresponding to a compliance 5% higher than the initial one [33].

**Fig. 9:**
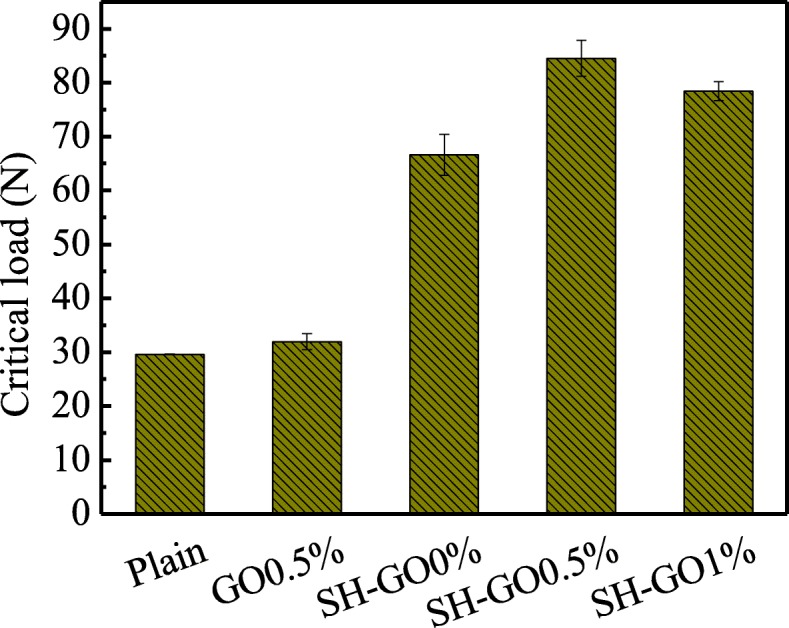
Critical load *P*_C_ for various specimens during DCB tests

Figure 9 presents the critical load *P*_C_ of the tested specimens. We can see that the *P*_C_ for the GO0.5% specimen is similar to the plain, which is far less than those of the other types of specimens. After the aluminum alloy was pre-treated by alkaline etching, the *P*_C_ of the SH-GO0% specimen is significantly increased, indicating an important role played by the surface treatment in the interfacial adhesion. It is worth noting that the critical load *P*_C_ for the SH-GO0.5% specimen is further increased when combining the alkali etching and addition of 0.5 wt% GO, and the highest obtained *P*_C_ is about 160% higher than those of the plain and the GO0.5% specimen, which indicates a possible synergic toughening effect between the surface treatment and GO interleaf. However, the *P*_C_ declines with further increase of the GO content (SH-GO1%), which could be attributed to the agglomeration of GO at a higher concentration.

**Fig. 10:**
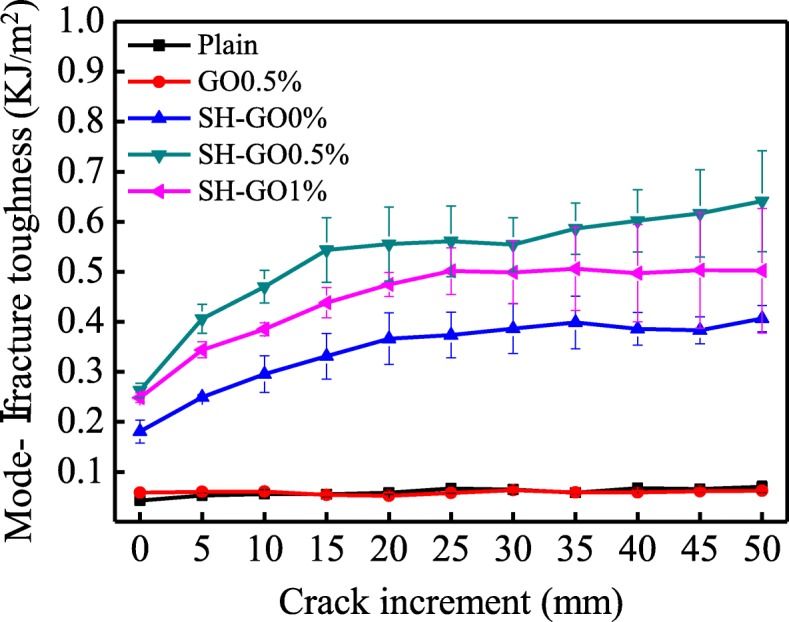
Comparison of the *R*-curves for various specimens during DCB tests

Figure 10 represents the mode-I fracture toughness as a function of crack growth increment ∆*a* (*R*-curve) for the tested samples. As can be seen, for the plain and GO0.5% specimen, the mode-I fracture toughness is independent of the crack growth increment ∆*a*, which also indicates the weak interfacial bonding between the degreased aluminum alloy and the glass fiber laminates. However, for the other types of the specimens, a typical fracture behavior can be observed, where the mode-I fracture toughness firstly increases with the crack growth, and then becomes stable due to the glass fiber bridging effect.

**Fig. 11:**
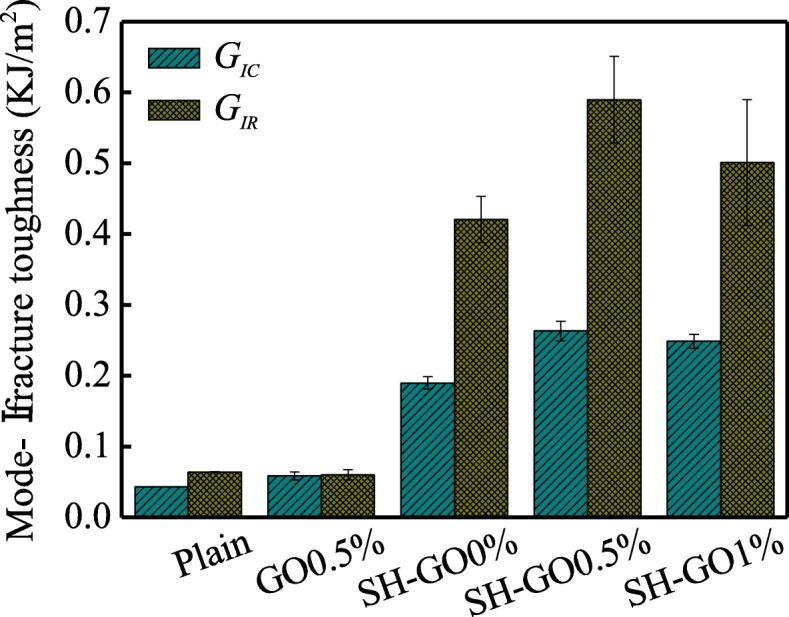
Comparison of the mode-I fracture toughness and resistance for various specimens

To better understand the effects of the surface treatment and GO interleaf on the interlaminar mechanical properties of the GFRP/Al laminates, the mode-I fracture toughness *G*_IC_ and fracture resistance *G*_IR_ are summarized in Fig. 11, where *G*_IC_ is the onset value on the *R*-curve and *G*_IR_ is the mean value of five points within the range of the crack extension ∆*a* from 20 to 40 mm. As can be seen from Fig. 11, there is no significant difference in *G*_IC_ and *G*_IR_ between the plain and the GO0.5% specimen. However, substantial increases of 225% and 600% in *G*_IC_ and *G*_IR_ for the SH-GO0% specimen can be observed when the Al alloy plates were treated with the alkaline chemical etching. This enhancement is due to the fact that the surface morphology and chemistry as well as the wettability of the Al alloy plates are improved by the alkaline etching treatment as described in the “Mode-I interlaminar fracture toughness” section. For the synergistic toughening specimens (SH-GO0.5%, and SH-GO1%), both the *G*_IC_ and *G*_IR_ are much higher than those of the specimens toughened with surface treatment only (SH-GO0%) or GO interleaf only (GO0.5%), which may be attributed to the synergistic effects of the surface treatment (enhanced interfacial adhesion) and the GO interleaf (toughened epoxy matrix). The maximum *G*_IC_ and *G*_IR_ observed in SH-GO0.5% specimens are 263 J/m^2^ and 590 J/m^2^, respectively, which are about 510% and 820% higher than those of the plain, respectively.

### Mode-II Interlaminar Fracture Toughness

**Fig. 12:**
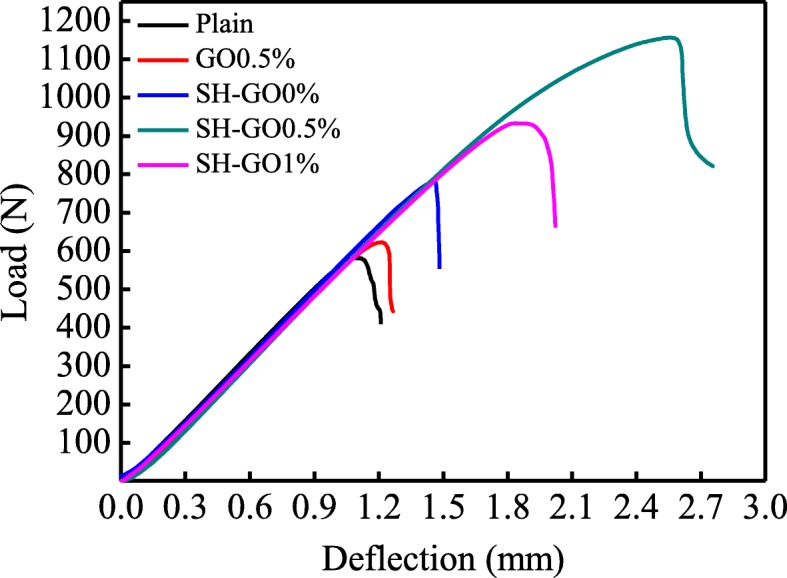
Representative load-deflection curves for different specimens during ENF tests

**Fig. 13:**
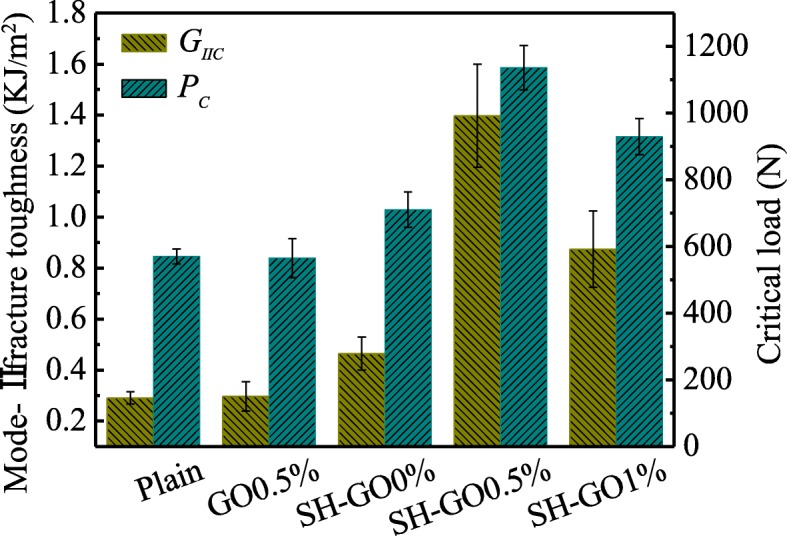
Comparison of mode-II fracture toughness *G*_IIC_ and critical load *P*_C_ for various specimens during ENF tests

Mode-II load-deflection curves of ENF specimens are shown in Fig. 12. Typically, the load-deflection curves show a linear response in the initial stage, and then a nonlinear response up to the maximum load, followed by an abrupt drop in the final stage. Figure 13 shows the critical load *P*_C_ and mode-II interlaminar fracture toughness *G*_IIC_ of the tested specimens calculated from the load-deflection profiles. It should be noted that the criterion for defining the critical load *P*_C_ for the ENF specimens is similar to that of the DCB specimens. We can see that both *G*_IIC_ and *P*_C_ of ENF specimens have the same tendency with that of DCB specimens. The maximum values of the mode-II fracture toughness and the critical load are observed in the specimen of SH-GO0.5%, which are 381% and 99% higher than those of the plain specimen, respectively.

### Observation of the Fracture Morphology

To further reveal the toughening mechanisms, the fracture morphologies of the tested GFRP/Al laminates were observed by SEM.

**Fig. 14:**
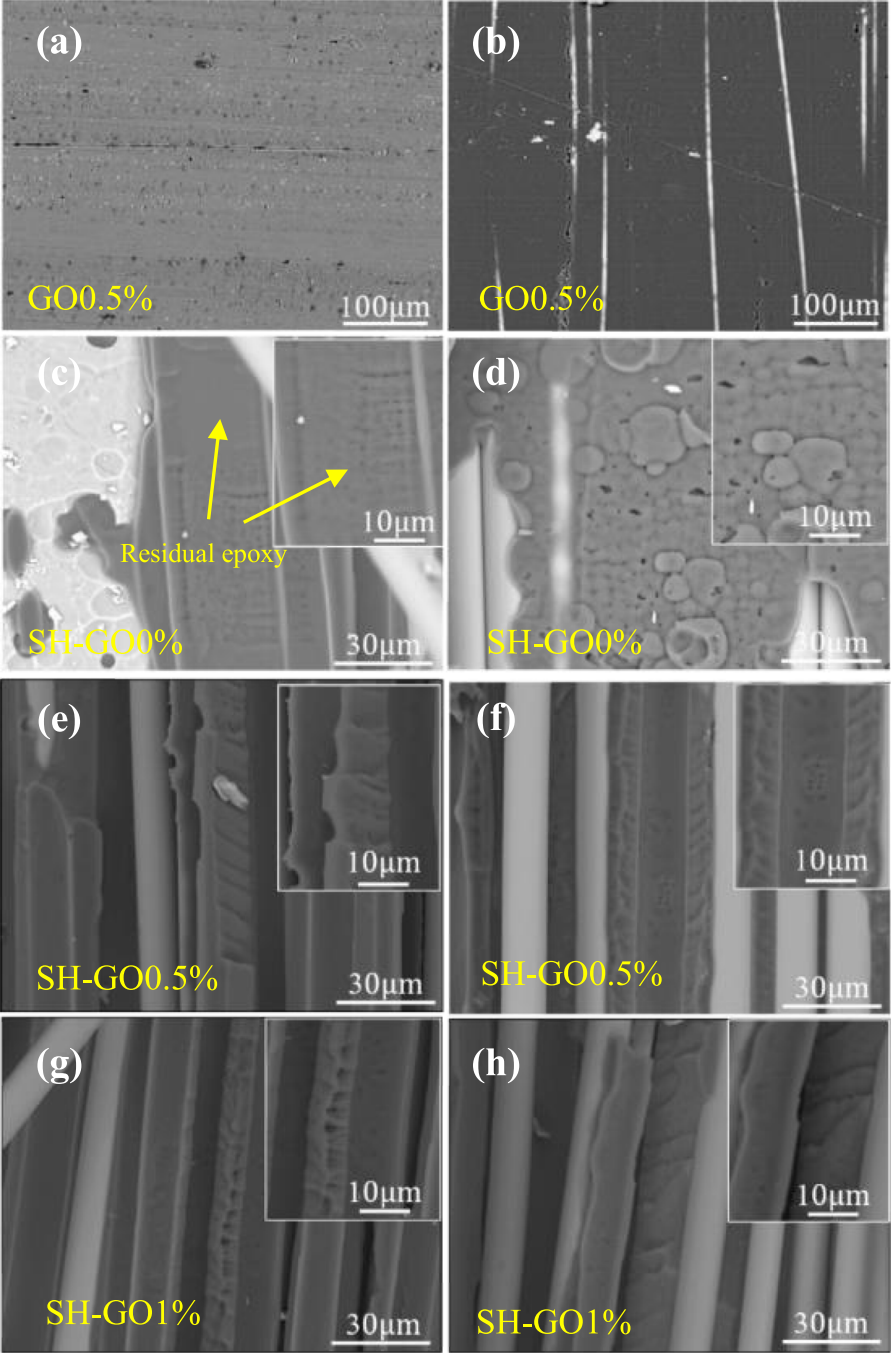
Fracture surfaces of mode-I GFRP/Al laminates. **a, b** GO0.5%. **c**, **d** SH-GO0%. **e**, **f** SH-GO0.5%. **g**, **h** SH-GO1% (left, the Al side; right, the composite side)

Figure 14 shows the fracture surfaces of the GO0.5%, SH-GO0%, SH-GO0.5%, and SH-GO1% specimens after DCB tests. With regard to the GO0.5% specimen (see Fig. 14a and b), the fracture surface has a smooth appearance, without any visible glass fiber or epoxy resin attached on the surface of the Al alloy plates. The failure type of the GO0.5% specimen is adhesive failure. As for the SH-GO0% specimen (see Fig. 14c and d), some broken fibers and epoxy resin attached on the surface or embedded in the micro-voids can be observed, which indicates that alkali etching could promote mechanical interlocking between the Al alloy plate and polymer matrix and then improve the interfacial bonding between them. The failure type of the SH-GO0% specimen is a combination of cohesive and adhesive. Cohesive failure caused by the debonding of resin molecules may consume more energy compared with interfacial failure [19], indicating the SH-GO0% specimen has a higher mode-I fracture toughness compared to the GO.5% specimen. With regard to the SH-GO0.5% and SH-GO1% specimens (see Fig. 14e–h), a more irregular and rougher fracture morphology can be observed, which will create a larger fracture area, and require a higher driving force and energy. The failure type of the SH-GO0.5% and SH-GO1% specimens is almost cohesive failure, which indicates that the addition of GO interleaf can further improve the interlaminar fracture toughness of the GFRP/Al laminates with the surface treatment. Possible reasons are as follows: Due to its excellent mechanical properties, GO can effectively improve the toughness of the epoxy resin by inducing the crack deflection and crack bridging effect [30], which commonly requires a higher driving force and higher fracture energy. Meanwhile, the functional groups on the surface of the GO sheets will contribute to the strong interfacial bonding between GO and epoxy resin, which may consume more energy during the process of pulling out of GO from epoxy matrix. Moreover, the addition of GO increases the reactive functional groups of the resin matrix [39, 40]. Therefore, the mode-I fracture toughness for the SH-GO0.5% and SH-GO1% specimens are further increased compared to the SH-GO0% specimen.

Based on the above analysis, the synergistic effect of the surface treatment and GO-epoxy interleaf on the improvement of mode-I interlaminar fracture toughness of Al/GFRP laminates has been demonstrated. However, excessive GO may have a negative effect on the fracture toughness. Because the aggregation of GO may cause stress concentration and reduce the toughness of the epoxy (see Fig. 3), the mode-I fracture toughness of SH-GO1% is lower than that of the SH-GO0.5% specimen.

**Fig. 15:**
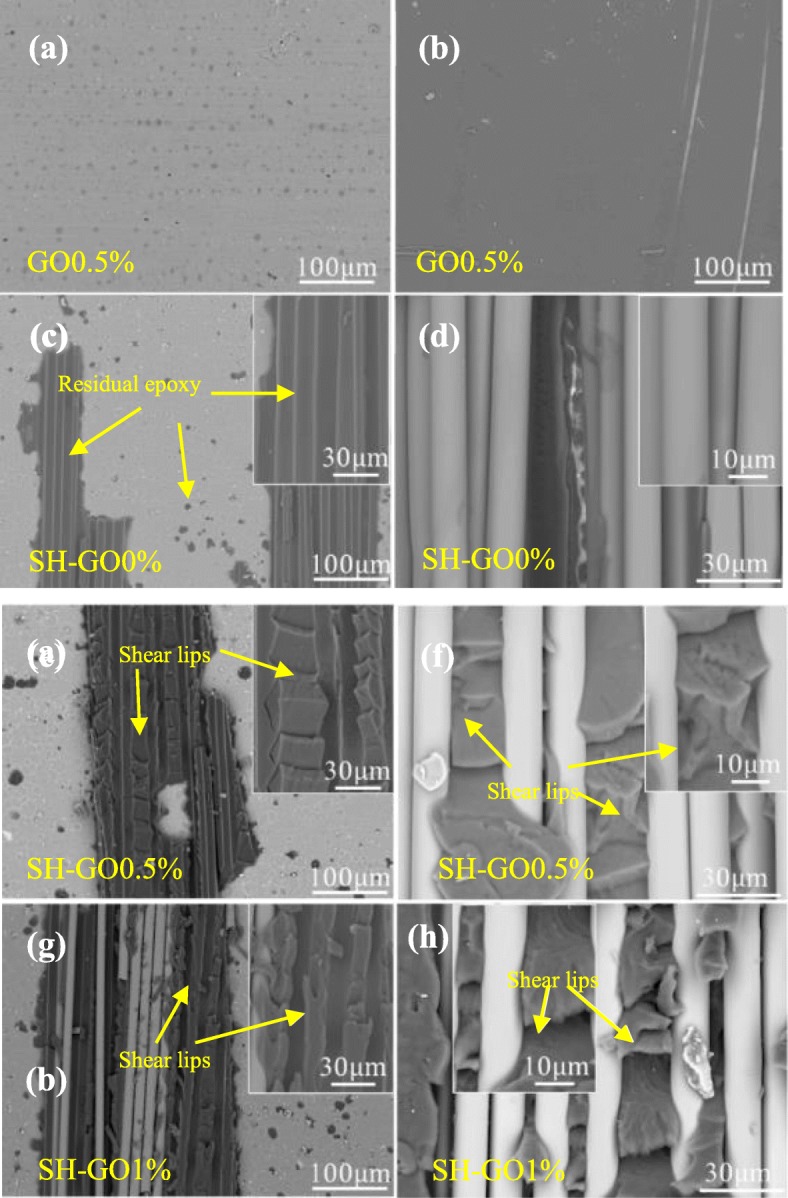
Fracture surfaces of the mode-II GFRP/Al laminates. **a**, **b** GO0.5%. **c**, **d** SH-GO0%. **e**, **f** SH-GO0.5%. **g**, **h** SH-GO1% (left, the Al side; right, the composite side)

SEM was also employed to investigate the ENF fracture surfaces of the tested specimens. For the GO0.5% specimen (Fig. 15a and b), the fracture surfaces on the Al plate and GFRP side are relatively smooth, which is similar to the DCB fracture morphology of the GO0.5% specimen. Broken fibers and residual epoxy adhered on the surfaces of the Al plate can be observed for the SH-GO0% (Fig. 15c), SH-GO0.5% (Fig. 15e), and SH-GO1% specimens (Fig. 15g), which implies the occurrence of cohesive failure and higher fracture toughness compared to that of the GO0.5% specimen. In addition, there are a lot of typical shear lips on the surfaces of the Al plates and composite sides for the SH-GO0.5% and SH-GO1% specimens, indicating an increased damage zone and a larger plastic deformation, which may lead to a higher mode-II fracture toughness than that of the SH-GO0%. Furthermore, the aggregation of GO may also be the main reason for the lower mode-II fracture toughness of the SH-GO1% specimen compared to that of the SH-GO0.5% specimen.

## Conclusions

In this study, surface treatment, e.g., alkali etching, and GO-epoxy interleaf were combined to synergistically improve the interlaminar mechanical properties of the Al/GFRP laminates. The DCB and ENF results demonstrate that the specimens with the alkali etching treatment and the GO0.5%-epoxy interleaf possess the highest mode-I and mode-II interlaminar fracture toughness, which are 510% and 381% higher than those of the plain specimen, respectively. In addition, different characterization technologies were employed to investigate the surface properties of the Al plates and the fracture surface of the tested laminates to uncover the synergistic toughening mechanisms.

## Data Availability

The datasets supporting the conclusions of this article are included within the article.

## Abbreviations

Al: Aluminum
FRPs: Fiber-reinforced plastics
GFRP/Al laminates: Glass fiber-aluminum laminates
GFRP prepregs: Glass fiber prepregs
GO: Graphene oxide
DCB: Double cantilever beam test
ENF: End-notched flexure test
XPS: X-ray photoelectron spectroscopy
SEM: Scanning electron microscope
TEM: Transmission electron microscopy
AFM: Atomic force microscopy
FMLs: Fiber metal laminates
NaOH: Sodium hydroxide
DMF: *N*,*N*-dimethylformamide
JIS: Japanese Industrial Standards
*P*-COD: Load and crack opening displacement
*G*_IC_: Mode-I fracture toughness
*G*_IR_: Mode-I fracture resistance
*G*_IIC_: Mode-II fracture toughness
*P*_C_: Critical load
γ-Al_2_O_3_: γ-Aluminum oxides
SH: Alkali etching treatment of Al plates
*R*_*a*_: The arithmetic average deviation of the profile
*R*_*q*_: The root mean square roughness
*R*_*z*_: The ten-point height of irregularities
